# Persistence of Gender Related-Effects on Visuo-Spatial and Verbal Working Memory in Right Brain-Damaged Patients

**DOI:** 10.3389/fnbeh.2016.00139

**Published:** 2016-06-28

**Authors:** Laura Piccardi, Alessandro Matano, Giovanni D’Antuono, Dario Marin, Paola Ciurli, Chiara Incoccia, Paola Verde, Paola Guariglia

**Affiliations:** ^1^Life, Health and Environmental Science Department, University of L’Aquila, L’AquilaItaly; ^2^IRCCS Fondazione Santa Lucia, Neuropsychology UnitRome, Italy; ^3^IMFR Gervasutta HospitalUdine, Italy; ^4^Aerospace Medicine Department, Italian Air Force Experimental Flight CentrePratica di Mare, Italy; ^5^Dipartimento Scienze dell’Uomo e della Società, UKE UniversityEnna, Italy

**Keywords:** sex differences, visuo-spatial working memory, active and passive processes, verbal working memory, short-term memory, right brain-damaged patients, cerebral lesions, cognitive reserve

## Abstract

The aim of the present study was to verify if gender differences in verbal and visuo-spatial working memory would persist following right cerebral lesions. To pursue our aim we investigated a large sample (*n*. 346) of right brain-damaged patients and healthy participants (*n*. 272) for the presence of gender effects in performing Corsi and Digit Test. We also assessed a subgroup of patients (*n*. 109) for the nature (active vs. passive) of working memory tasks. We tested working memory (WM) administering the Corsi Test (CBT) and the Digit Span (DS) using two different versions: forward (fCBT and fDS), subjects were required to repeat stimuli in the same order that they were presented; and backward (bCBT and bDS), subjects were required to repeat stimuli in the opposite order of presentation. In this way, passive storage and active processing of working memory were assessed. Our results showed the persistence of gender-related effects in spite of the presence of right brain lesions. We found that men outperformed women both in CBT and DS, regardless of active and passive processing of verbal and visuo-spatial stimuli. The presence of visuo-spatial disorders (i.e., hemineglect) can affect the performance on Corsi Test. In our sample, men and women were equally affected by hemineglect, therefore it did not mask the gender effect. Generally speaking, the persistence of the men’s superiority in visuo-spatial tasks may be interpreted as a protective factor, at least for men, within other life factors such as level of education or kind of profession before retirement.

## Introduction

The clearest evidence of gender-related differences emerges in spatial abilities (Hyde, 2005). However, differences are larger for some spatial abilities than for others and, on average, men perform better than women (Weiss et al., 2003; Lawton, 2010). One of the most consistent findings regarding gender cognitive differences relies on tasks tapping spatial rotation and dynamic transformations of material in visuo-spatial memory (Coluccia and Iosue, 2004; Millet et al., 2009; Lawton, 2010; Verde et al., 2013). Male advantage in visuo-spatial abilities emerges very early in life (Geiser et al., 2008), remaining stable in middle-aged and elderly adults (De Frias et al., 2006). The role of sexual hormones has been proposed to partially explain these differences (Pompili et al., 2012) and it is supported by evidence thereby hormonal changes during pregnancy affect women’s performances on some visuo-spatial tasks (Brett and Baxendale, 2001; Piccardi et al., 2013a, 2014b). A cross-sectional study of women at three stages of the menopause transition, Berent-Spillson et al. (2012), found an effect of menopause status, independent of age, on aspects of verbal but not visual or executive cognitive function. Menopause stage groups differed in neuropsychological measures. In particular, the authors found differences in verbal fluency, but not visual memory, between the groups, suggesting that declines in verbal ability are an effect of the menopausal transition independent of aging. However, the presence of gender effects may also be attributed to differences in cognitive strategies, socio-environmental factors, and training effects (Lawton, 2010). Studies about gender differences in memory, evidenced that women are better than men in remembering past episodes (Wang, 2013) and in general in verbal episodic memory, due to a female advantage in verbal production (Herlitz and Rehnman, 2008) Women are also better in remembering to do an event-based task (Palermo et al., 2016). However, the advantage of men over women emerges in visuo-spatial working memory (Orsini et al., 1986, 1987; Capitani et al., 1991; Piccardi et al., 2008b, 2013b). Several authors proposed a distinction between passive and active processing (Logie, 1995; Cornoldi and Vecchi, 2003; Coluccia and Iosue, 2004). Specifically, passive storage (i.e., when the task requires the simple recollection of previously acquired information) or active processing (i.e., when the task requires integration and manipulation of information to produce an output substantially different from the original input) play an important role in bringing to light the presence of gender-related effects (Vecchi and Richardson, 2000). Generally speaking, men have an advantage over women in tasks requiring active manipulation of mentally generated images. This is explained in terms of different cognitive strategies they use (Grön et al., 2000). Women tend to select less efficient, verbally mediated (analytic), strategies (Lawton, 2010), whereas men use a more efficient, spatially mediated, strategy (Heil and Jansen-Osmann, 2008). The use of different strategies in solving visuo-spatial tasks can be observed also in f-MRI. Grön et al. (2000) found that women engage the right parietal and the right prefrontal cortex when performing a virtual maze task. Conversely, men address this task by recruiting the left hippocampus. A possible explanation of the activity of the prefrontal cortex observed in women, could be a higher performance of working memory engaged in holding landmark cues “online” during the virtual navigation. On the other hand, the left hippocampus activity observed in men may be due to the processing of multiple geometric cues during the navigation in the virtual maze. This interpretation is in line with the hypothesis advanced by Coluccia and Iosue (2004). They hypothesized that a women’s disadvantage occurs when the visuo-spatial working memory load increases, especially in tasks requiring active processing of visuo-spatial information.

An inverse pattern of these differences has been described for verbal working memory and, more in general, for verbal tasks (i.e., verbal fluency, synonym-generation: Kimura and Harshman, 1984; Kimura, 1999; Halpern, 2000; Lewin et al., 2001; Maitland et al., 2004). Most of the studies report that women outperform men but this gender difference seems to decrease with aging. Ryan et al. (2008) suggested that effects of gender on verbal learning tasks become insignificant once age and education levels are considered. A meta-analysis by Lynn and Irwing (2008) about Digit Span in children and in adults suggests that, when analyzing gender differences, it is necessary to include some moderators of the size of the effect (i.e., the age). Specifically, these authors found a small female advantage of 0.134 Cohen’s d for children and adolescents and a small male advantage of 0.116 Cohen’s d among adults. Thus, in agreement with what previously observed about visuo-spatial working memory, this meta-analysis highlights that gender differences in verbal working memory are influenced by several variables. The existence of a male advantage for visuo-spatial memory and of a female advantage for verbal memory is supported by most of, but not all, the studies (Lynn and Irwing, 2008).

In the past few decades, several studies have shown that factors such as high levels of education, occupational complexity, and/or pre-morbid intelligence were associated with lower levels of cognitive impairment in neurological conditions such as Alzheimer’s Disease (Roe et al., 2010), stroke (Ojala-Oksala et al., 2012) and in patients with traumatic brain injury (Grafman, 1986; Kesler et al., 2003). Stern (2012) expands the initial definition of cognitive reserve, considering a further distinction between two types of “reserve”: the brain reserve and the cognitive reserve. The first refers to differences in brain structures which may result in increasing the brain’s tolerance to disease. The second refers to individuals’ differences in cognitive performances in spite of brain damages.

In this direction, an individual less explored factor is gender and the resilience to brain damages.

For this reason, in the present study, we investigated whether gender differences persist also in patients suffering from a right brain lesion involving cortical and subcortical structures of the parietal lobe. We hypothesized that visuo-spatial working memory performance should be more greatly affected by the cerebral lesion compared to verbal working memory. We also considered the presence/absence of hemineglect (i.e., a common neurological condition consequence of right brain damage, whereby patients fail to be aware of stimuli or to detect them in the left space) in our sample. Pisella et al. (2004) demonstrated that the deficits in working memory in patients with neglect resulting from parietal lesions are specific to the spatial representation of stimuli. Furthermore, some studies suggest that performance in Corsi Test is worse in patients with right hemisphere damage, but this can be dissociated from the presence of visual field deficits and neglect (De Renzi et al., 1977; Kessels et al., 2002, 2008). Of course, task performance can be affected by the presence of neglect if there is a failure to attend to leftward locations in the tapped sequence (Malhotra et al., 2005). Chechlacz et al. (2014) found that the neural substrates of spatial working memory in the examined patients overlapped with lesion locations within the right posterior parietal cortex, a region also linked to the symptoms of neglect (Heilman et al., 1983; Vallar and Perani, 1986; Husain et al., 2001; Wojciulik et al., 2001; Mort et al., 2003; Della Sala et al., 2004; Mannan et al., 2005; Committeri et al., 2007; Malhotra et al., 2009; Corbetta and Shulman, 2011). Although, the performance on the Corsi test may be affected by the failure to attend to contralesional stimuli directly as a result of neglect, not all patients with hemineglect show spatial working memory deficits when performing Corsi Test (e.g., Mannan et al., 2005; Piccardi et al., 2008a).

For investigating visuo-spatial working memory, we used the Corsi test (CBT), one of the most widely used visuo-spatial working memory tools. It involves reproducing a sequence shown by the examiner on a set of 9 identical wooden blocks positioned on a board. The level of difficulty is increased by adding blocks to the sequence to be remembered. The examiner stops when the participant is unable to reproduce at least two out of three trials of a given length. In the forward Corsi test (fCBT) the participant is asked to reproduce the block-sequence in the given order. In the backward Corsi Test (bCBT) the participant needs to reverse the order of the block-sequence. The final score (Corsi Span) is given from the longer sequence the participant is able to reproduce. The CBT is a test often used to observe gender differences (Orsini et al., 1986, 1987; Capitani et al., 1991; Piccardi et al., 2008b, 2013a, 2014a; Verde et al., 2015), even if the magnitude and consistency of these differences in spatial memory have been widely questioned and seem to be procedure-dependent (Berch et al., 1998; Piccardi et al., 2011, 2014a). For example, Shah et al. (2013) found sex differences in the standard CBT, but they did not find gender differences in a computerized version of the test. For this reason, in the present study we used the standard CBT and we evaluated both passive storage and active processing of visuo-spatial information.

Furthermore, we also investigated verbal working memory by means of Digit Span. The Digit Span (DS) is one of the most famous verbal working memory tests. Participants are asked to repeat a series of digits. In the forward digit-span task (fDS) the participant is asked to enter the digits in the given order. In the backward digit-span (bDS) task the participant needs to reverse the order of the numbers. If the participants are able to repeat one out two sequences correctly, they are given a longer list. The length of the longest list a participant can remember is that person’s digit span.

We did not expect to find any effects related to the brain lesion in verbal working memory, since we investigated it only in right brain-damaged patients. The issue of gender differences in visuo-spatial working memory has been poorly investigated in patients with right brain lesions. Since neuroimaging studies demonstrated the role of critical regions (i.e., hippocampal formation, right parietal and prefrontal cortex) (Grön et al., 2000; Nemmi et al., 2013), we investigated whether gender differences in visuo-spatial active manipulation can still be evidenced in right brain-damaged patients and whether they are linked to the nature of the stimuli to be processed (visuo-spatial vs. verbal) or to the type of processing required (active processing vs. passive storage).

In order to verify the hypothesis, a sub-group of patients performed two different versions of the Corsi test (Corsi, 1972) and the Digit Span (Orsini et al., 1987): a) the forward version (fCBT and fDS), assessing passive working memory, and b) the backward version (bCBT and bDS), assessing active working memory. We expected to find a protective effect of gender in male patients about performances on visuo-spatial working memory tests.

## Materials and Methods

### Participants

All patients admitted to IRCCS Fondazione Santa Lucia (Rome) in the last 5 years (2010–2015) have been included in this study and their medical records have been analyzed on the basis of a gender perspective. Specifically, we analyzed data concerning 507 brain-damaged patients with sequelae strokes who showed no comprehension deficits or mental decay on the neuropsychological assessment at admission. Exclusion criteria were as follows: left handedness, a history of multiple cerebrovascular accidents, general cognitive decay, previous neurological or psychiatric disorders and use of any drugs and/or any supplements, vitamins, hormones (including herbal) that may significantly affect cognitive performance. As a consequence, 161 patients were excluded from the statistical analysis. The studied sample included 346 right brain-damaged patients: 146 women (Women Patients = WP) and 200 men (Men Patients = MP). A subgroup of 109 right brain damaged patients performed the Corsi test both forward and backward.

In our sample, 109 patients suffered from hemineglect as diagnosed through the standard battery for evaluating the neglect syndrome (Pizzamiglio et al., 1989). This was equally distributed between male and female patients.

A control group of 272 healthy participants were matched for age and gender with the right brain-damaged patient group. Specifically, we recruited 148 Women Healthy Participants (WHP) and 124 Male Healthy Participants (MHP). Demographic and clinical data were reported in **Table 1**, as well as statistical analysis demonstrating that the two patient groups did not differ for stroke onset, and all groups did not differ for age and educational level (**Table 1**). All healthy participants performed Raven’s Matrices (Raven, 1938) to exclude the presence of problems in visuo-spatial reasoning and answered a questionnaire in which they were asked to indicate any previous or current diseases (including substance abuse or dependence). All healthy participants were also asked if they had had adequate sleep or had not recently traveled across time zones, had normal or corrected-to-normal vision and had not drunk coffee or smoked cigarettes before performing the tests.

**Table 1:** Means ± standard deviations (range) of age, level of education, stroke onset; percentage of hemineglect in the two groups of patients and healthy participants.

	WP	MP	WHP	MHP	One-way ANOVA
Age (years)	65.64 ± 14.98 (24–87)	62.37 ± 13.79 (20–88)	63.57 ± 6.34 (55–80)	62.74 ± 6.18 (55–80)	*F*_(3.614)_ = 2.53; *p* = 0.06
Education (years)	10.71 ± 4,69 (3–19)	11.24 ± 4.22 (3–19)	9.97 ± 3.90 (3–18)	11.23 ± 4.23 (5–18)	*F*_(3.614)_ = 2.99; *p* = 0.03
Stroke Onset (days)	86.21 ± 317.45 (8–2920)	51.65 ± 102 (8–1095)	–	–	*F*_(1.331)_ = 2.01; *p* = 0.16
Hemineglect	33%	30%	–	–	
Raven	21.23 ± 6.52 (5–36)	23.18 ± 7.03 (8–36)	31.83 ± 11.37 (10–60)	35.43 ± 10.53 (10–60)	

*Means and SD of Raven score are also shown (patients max Raven score = 36; healthy participants max score = 60; all participants are in the normal range).*

*WP = women patients; MP = men patients; WHP = women healthy participants; MHP = men healthy participants.*

The study protocol, which was in accordance with the ethical principles of the Declaration of Helsinki, was approved by the local ethics committee (I.R.C.C.S. Fondazione Santa Lucia of Rome, Italy). All patients and healthy participants were compos mentis and signed a written consent form before taking part in the experimental testing.

### Neuropsychological Assessment

All patients were submitted to an extensive neuropsychological assessment to investigate their orientation in time and space, personal orientation (Spinnler and Tognoni, 1987), language functions (Ciurli et al., 1996), visuo-spatial and verbal short-term and working memory (Spinnler and Tognoni, 1987), long-term verbal memory (Spinnler and Tognoni, 1987), abstract and/or verbal reasoning (Raven, 1938; Spinnler and Tognoni, 1987), attention and agnosia (Spinnler and Tognoni, 1987). Patients’ performance on the neuropsychological tests was used to rule out general mental decay.

### Stimuli and Procedure

Visuo-spatial working memory was tested using the Corsi Test (CBT; Corsi, 1972). This test consists of 9 wooden blocks (4.5 cm × 4.5 cm) fixed on a board (30 cm × 25 cm) in a scattered array. On the experimenter’s side, the blocks are numbered for easy identification. The participants were seated facing the examiner on a height-adjustable office chair in front of the CBT board. The examiner tapped a sequence of blocks at the rate of 1 block per 2 s, and the participants had to reproduce the same sequence in (a) the same order in the fCBT and in (b) the reverse order in the bCBT. Sequences of increasing length (i.e., number of blocks starting from a 2-block sequence onward) were presented until the participants failed to reproduce 2 out of 3 trials of a given length.

Verbal working memory was tested using Digit Span (DS), a subtest of the Wechsler Intelligence scale (Wechsler, 1981; Orsini et al., 1987). Auditory DS was assessed using number sequences; two test items were presented at each sequence length. The participants had to recall the same sequence in (a) the same order in the fDS and in (b) the reverse order in the bDS. The number of correct items was recorded as a digit score and the digit span was calculated as the longest sequence of digits recalled correctly.

The participants were tested individually in a quiet hospital room with artificial lighting.

### Statistical Analyses

Statistical analyses were performed using SPSS (IBM SPSS Statistics 20). Descriptive analysis included means and standard deviations of participants scores of the different tests, subdivided in to four groups (WP, MP, WHP, and MHP). A correlation was run between demographic variables (age, gender, and education) and visuo-spatial and verbal working memory tasks (fCBT; bCBT; fDS, and bDS). To test the Gender effect on WM tasks, we ran a Multivariate ANOVA with four Groups (WP vs. MP and WHP vs. MHP) as between factors. Performances at the two tasks, taking into account passive processing and active storage of verbal and visuo-spatial information (fDS vs. fCBT vs. bDS vs. bCBT), were considered as within factors. Then we also performed separate ANOVA for visuo-spatial working memory and verbal working memory.

## Results

**FIGURE 1:**
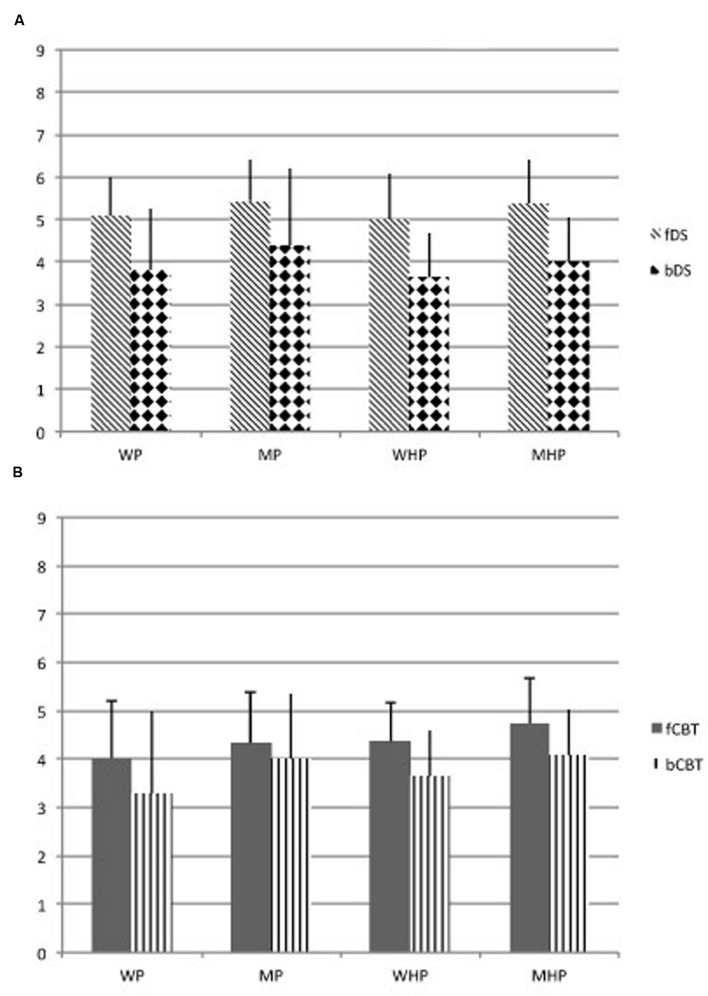
**(A)** Groups’ average performance and standard deviation of span (the longest list of items: digits) obtained in the Digit Span, taking into account active and passive processes. **(B)** Groups’ average performance and standard deviation of span (the longest list of items: blocks) obtained in the Corsi Test, taking into account active and passive processes. WP, women patients; MP, men patients; WHP, women healthy participants; MHP, men healthy participants; fDS, forward Digit Span; bDS, backward Digit Span; fCBT, forward Corsi Test; bCBT, backward Corsi Test.

Means and standard deviations of the whole sample subdivided in the four groups are shown in **Figure 1**.

Pearson correlations (with alpha level set at 0.05) were computed between demographic variables, performance on fDS, fCBT, bDS, and bCBT. Results show that all variables correlate (**Table 2**).

**Table 2:** Pearson correlations between demographic variables, performance on fDS, fCBT, bDS, and bCBT.

	Age	Education	fCBT	bCBT	fDS	bDS
Age		–0.22**	–0.24**	–0.34**	–0.3**	–0.29**
Education			0.19**	0.30**	0.42**	0.31**
fCBT				0.61**	0.30**	0.35**
bCBT					0.38**	0.45**
fDS						0.51**

**p = 0.01.

*fDS = forward Digit Span; bDS = backward Digit Span; fCBT = forward Corsi Test; and bCBT = backward Corsi Test.*

Multivariate ANOVA showed a main effect of Groups (*F*_(3,369)_ = 9.94, *p* = 0.001; Cohen’s *d* = 0.998; effect size [*r*] = 0.075) and Tasks (*F*_(3,1107)_ = 25.54, *p* = 0.001 Cohen’s *d* = 0.999; effect size [*r*] = 0.065), but did not show an interaction effect Groups x Tasks (*F*_(9,1107)_ = 0.35, *p* = 0.79; Cohen’s *d* = 0.119; effect size [*r*] = 0.003). A Bonferroni Test demonstrates that women and men healthy participants were significantly different (*p* < 0.01) as well as women and men right brain damaged patients (*p* < 0.01). Men (both healthy controls and patients) had significantly higher scores than women in all tests. Concerning the working memory tasks, fDS was significantly different from bDS (*p* < 0.01) and from fCBT (*p* < 0.01); likewise, fCBT was significantly different from bCBT (*p* < 0.01), but it did not differ from bDS (*p* = 0.845) (**Figures 1A,B**). In general, verbal working memory obtained higher scores than visuo-spatial working memory. In both tasks, all participants obtained higher scores in forward than backward tasks.

The univariate ANOVA on performance in Digit Span, which accounted for active vs. passive processes, showed differences for Groups (*F*_(3,606)_ = 10.09, *p* = 0.001; Cohen’s *d* = 0.998; effect size [*r*] = 0.048) and processes (*F*_(1,606)_ = 586.03, *p* = 0.001; Cohen’s *d* = 1; effect size [*r*] = 0.492), but not for the interaction Groups x DS’ processes (*F*_(3,606)_ = 1.96, *p* = 0.12; Cohen’s *d* = 0.506; effect size [*r*] = 0.010). A Bonferroni test performed on Groups showed that women and men healthy participants were significantly different (*p* < 0.01) as well as women and men patients (*p* < 0.01). Men of both groups performed better than women. Women and men healthy participants did not differ from women and men patients (*p* = 0.783). Another univariate ANOVA was performed on Groups and performances in CBT considering active and passive processes. This analysis showed a significant effect of Groups (*F*_(3,370)_ = 7.99, *p* = 0.001; Cohen’s *d* = 0.991; effect size [*r*] = 0.061) and CBT’s processes (*F*_(1,370)_ = 191.25, *p* = 0.001; Cohen’s *d* = 1; effect size [*r*] = 0.341), but it did not show any significant interaction Group x CBT’s processes (*F*_(3,370)_ = 1.08, *p* = 0.36; Cohen’s *d* = 0.293; effect size [*r*] = 0.009). A Bonferroni Test showed that women and men healthy participants were significantly different (*p* < 0.01) as well as women and men patients (*p* < 0.01). Men outperformed women in all tasks both in healthy participant and patient groups. Men and women healthy participants did not differ from men and women patients (*p* = 0.152).

Although, the presence of hemineglect was equally distributed between men and women patients (**Table 1**), we investigated whether patients suffering from hemineglect were significantly different from patients without neglect. To this purpose we run out a univariate ANOVA with Group (neglect vs. no-neglect) and CBT’s processes (fCBT vs. bCBT) as dependent variables. From this analysis emerges a significant difference for Groups (*F*_(1,100)_ = 31.58, *p* = 0.001; Cohen’s *d* = 1; effect size [*r*] = 0.240) and for CBT’s processes (*F*_(1,100)_ = 31.00, *p* = 0.001; Cohen’s *d* = 1; effect size [*r*] = 0.237), but it does not show any significant interaction between Groups x CBT’s processes (*F*_(1,100)_ = 0.07, *p* = 0.79; Cohen’s *d* = 0.058; effect size [*r*] = 0.001). Groups with neglect showed a lower performance on CBT both forward and backward. The active process (bCBT) is more difficult than passive one (fCBT).

## Discussion

In the present study, we investigated the hypothesis that gender differences in verbal and visuo-spatial working memory, referring to specific processes of the working memory system, namely the active ones, may persist also in right brain-damaged patients. This hypothesis has been tested by using two standard instruments (CBT and DS) that, by using the same stimuli but varying the instructions (“repeat in the same order”/“repeat in a backward order”), allow to assess both active and passive processes of working memory. The forward version of the tests involves the passive maintenance of visuo-spatial or verbal information, while the backward version requires an active manipulation of such information. We expected an effect of the right brain lesions only on the visuo-spatial working memory but not on the verbal working memory. Surprisingly, our results demonstrated the persistence of gender differences in performing CBT, regardless of the presence of visuo-spatial disorders. Most likely, this result emerged because the presence of hemineglect was equally distributed in the two groups, men and women. Indeed, the fact that the two groups were matched for the presence of this condition has allowed to observe gender-related effects. Our data are in line with the finding that performance on the Corsi Test could be affected by the presence of neglect if there is a failure to attend to leftward locations in the tapped sequence (Malhotra et al., 2005). However, the evidence that in some cases (Mannan et al., 2005; Piccardi et al., 2008a), in spite of hemineglect, performance in CBT is within average, demonstrates that the impairment in visuo-spatial working memory is probably also affected by the severity of hemineglect itself. Interestingly, patients did not differ from healthy participants in performing working memory tasks. This result demonstrates that, despite a right brain lesion, men still present a greater ability than women to operate manipulation on visuo-spatial information and to use this dynamically changing information to guide their performance. Differently from other studies (e.g., Millet et al., 2009), we found that men outperformed women both in active and passive processes. We found men superiority in performing not only fCBT and bCBT, but also in performing the fDS and bDS. This result seems to be in contrast with literature that reports women superiority in verbal memory tasks. This discrepancy could be a specific effect of the cerebral damage that in some way affects them. However, a recent study reported that midlife men showed better performance on digit span backward compared with women (Jacobs et al., 2016). On one hand, one of the most consistently observed cognitive changes in midlife women is within the domain of verbal learning and memory (Berent-Spillson et al., 2012; Epperson et al., 2013). In our opinion, some type of the higher or lower male or female frequencies could be ascribed to the evidence that men have a higher level of education than women. Indeed, as reported in the meta-analysis by Lynn and Irwing (2008) on Digit Span, the level of education highly affects performances, making gender differences more evident in the participants with lower or absent education, or less evident increasing the education level. For all participants passive storage is easier than active processing. This is predictable since in backward CBT and DS, attentional resources are being increased with the necessity to reproduce the sequences in reverse order (Vandierendonck et al., 2004). In general, backward working memory tasks are assumed to require a higher degree of active manipulation than the forward working memory tasks. It is possible that the observed gender effects both in active and passive components are due to the age of our sample. Indeed, Park et al. (2002) described a working memory decline due to the neural changes in local gray and white matter (e.g., Rabbitt et al., 2008; Kennedy and Raz, 2009; Montembeault et al., 2012). These changes affect the frontal lobe (Raz et al., 1997; Resnick et al., 2003; Raz, 2008). In particular, Shanmugan and Epperson (2014) reported changes in frontally mediated executive functions (including working memory) that could explain a decrease of both passive and active components. De Frias et al. (2006) found that the magnitude of gender-related effects on visual working memory seems to be almost equal in middle-aged and old adults. Indeed, midlife and old men and women are the most numerous in our sample. Pauls et al. (2013) highlight that very little is known about how gender-specific advantages or disadvantages in memory performance are related to different ages in terms of magnitude of the effects. Moreover, it is still unclear how to establish when working memory begins to decline during adulthood. It is known that Corsi Block Tapping test’s performance is affected by aging (Orsini et al., 1986; Myerson et al., 1999). Cansino et al. (2013) found that between 41 and 70 years of age, men outperformed women in visuo-spatial tasks and, between 41 and 50 years of age, they outperformed women in verbal tasks. Despite, literature suggestions that women show a disadvantage in high-difficulty tasks (e.g., Vecchi and Girelli, 1998; Cattaneo et al., 2006), we failed in differentiating performances between active and passive tasks. However, it is possible that this lack of effect may be due to the type of working memory task performed by the participants, as well as to the procedure used in administering the task itself (e.g., Berch et al., 1998). A limitation of the present study could be to have included participants with a mean ages >60 years old. This age range leads to the assumption that most of the women patients were in post-menopausal period, which might alter their hormonal structure and also cognitive functions. However, even if this is a really interesting hypothesis, the absence of any hormonal data limits the possibility to hypothesize that our results are affected by hormonal changes as implied by the participants’ age.

However, preserved gender-related effects in neurological patients is an interesting finding because, as suggested by Millet et al. (2009) for Alzheimer Disease, the presence of gender differences may be considered in the context of the “cognitive reserve” hypothesis (Stern, 2006). As a consequence, beside lifestyle factors (i.e., level of education, occupational attainment, leisure activities), gender could prove to be another protective factor when people develop a neurological disorder.

## Author Contributions

Conceived and designed the experiment LP and PG. Performed the experiment and analyzed the data LP, AM, GD, DM, PC, CI, PV, and PG. Wrote the paper LP and PG.

## Conflict of Interest Statement

The authors declare that the research was conducted in the absence of any commercial or financial relationships that could be construed as a potential conflict of interest.
